# Coding-Complete Sequences of Barley Virus G Isolates from Australia, Obtained from a 34-Year-Old and a 1-Year-Old Sample

**DOI:** 10.1128/MRA.01292-19

**Published:** 2019-11-21

**Authors:** N. Nancarrow, S. Maina, L. Zheng, M. Aftab, A. Freeman, W. M. Kinoti, B. Rodoni, P. Trębicki

**Affiliations:** a Agriculture Victoria, Horsham, Victoria, Australia; b Agriculture Victoria, AgriBio, Bundoora, Victoria, Australia; DOE Joint Genome Institute

## Abstract

Coding-complete sequences of two barley virus G isolates from Australia were obtained from a 34-year-old oat sample (isolate Aus8) and a 1-year-old barley sample (isolate Aus17N). The Aus8 and Aus17N isolates share 96.3% nucleotide identity with each other and 95.7 to 95.8% nucleotide identity with the South Korean isolate Uiseong.

## ANNOUNCEMENT

Barley virus G (BVG) belongs to the genus Polerovirus (family *Luteoviridae*) and most closely resembles *Maize yellow dwarf virus* RMV (MYDV-RMV) (1). It was first reported in South Korea in 2016 (1) and was reported in Australia for the first time in 2019 (2). One BVG-positive barley plant sample (Aus17N) collected in Horsham, Victoria, Australia, in 2018 and studied using partial sequencing (2) was subjected to high-throughput sequencing (HTS), along with 11 other unrelated plant virus samples. Total RNA was extracted using the Qiagen RNeasy plant minikit with modified lysis buffer (3, 4) and treated with RNase-free DNase (Invitrogen). All quality control checks were done, and libraries were prepared using an Illumina TruSeq stranded total RNA kit with the Ribo-Zero plant library preparation kit, as previously described (5–7). The 12 libraries were pooled and sequenced using a MiSeq v3 kit (Illumina) with 2 × 251 cycles of paired-end (PE) reads.

In addition, an oat plant sample (Aus8) infected with a virus thought to be *Barley yellow dwarf virus* was collected in Melbourne, Victoria, in 1985 and desiccated on calcium sulfate (CaSO_4_). Total RNA was extracted using a Qiagen RNeasy plant minikit with a modified lysis buffer (3, 4) and tested positive for MYDV-RMV by reverse transcription-PCR (RT-PCR) (8). The 34-year-old sample was then subjected to HTS, along with 9 other unrelated plant virus samples. The total RNA extract was treated with RNase-free DNase (Invitrogen), and quality control checks were done (5). The 10 libraries were prepared using a NEBNext Ultra RNA library prep kit for Illumina (NEB), according to the manufacturer’s instructions, and were pooled and sequenced using a MiSeq v3 kit (Illumina) with 2 × 251 cycles of PE reads.

The PE raw reads (3,032,170 for Aus17N and 2,391,640 for Aus8) were trimmed using Trim Galore v0.4.4 (9), as previously described (10). *De novo* assembly of the resulting trimmed reads (3,001,650 for Aus17N and 2,353,020 for Aus8) was performed using the metaSPAdes v3.13.0 genome assembler (11), with default settings. In addition, reference mapping was done with Bowtie 2 v2.3.4.2 (12), with default settings, and mapped 55,549 reads to Aus17N and 11,774 reads to Aus8 with a mean coverage of 369 to 1,337×.

The resulting contigs of interest were imported into Geneious v2019.2.1 (13) and aligned with reference sequences using MUSCLE (14). A contig that closely matched BVG was obtained from each sample. The Aus17N and Aus8 sequences were 5,574 bp and 5,573 bp long, respectively, while the full-length BVG genome is 5,620 bp long (1). The GC content of both Aus17N and Aus8 was 50%. Open reading frames were predicted and annotated using Geneious, as previously described (10).

Sequence alignment showed that the Aus17N and Aus8 sequences shared 96.3% nucleotide (nt) identity with each other, and a BLASTN search (15) of the NCBI nucleotide (nr/nt) collection revealed that Aus17N and Aus8 resembled the South Korean BVG isolate Uiseong (GenBank accession number LC259081) at 95.8% and 95.7% nt identity, respectively. However, despite the 34-year-old isolate (Aus8) testing positive for MYDV-RMV by RT-PCR, Aus17N and Aus8 shared only 78.3% and 78.1% nt identity with the closest matching MYDV-RMV isolate (GenBank accession number MH205607), and no contigs obtained from either sample matched MYDV-RMV more closely than BVG.

These are the first coding-complete sequences of BVG from Australia. Additionally, the coding-complete sequence from sample Aus8 is the oldest reported BVG sequence to date, indicating that although BVG was first reported in 2016, it has been present in Australia much longer. The BVG genome information obtained will be used to improve its detection and differentiation from closely related viruses such as MYDV-RMV in cereal crops using molecular methods in the future.

### Data availability.

The sequences of the two Australian BVG isolates described here were deposited in DDBJ/EMBL/GenBank under accession numbers LC500835 and LC500836. The raw sequence data were deposited in the SRA under BioProject number PRJNA575278 and under BioSample numbers SRX6941552 and SRX6941553.
